# Evaluation of Clinical Outcomes and Treatment Complications in Hairy Cell Leukemia: A Single‐Center Retrospective Analysis

**DOI:** 10.1002/cnr2.70356

**Published:** 2025-10-01

**Authors:** M. Garcia Fasanella, A. Mozos, J. Briones, J. F. Nomdedeu, S. Novelli

**Affiliations:** ^1^ Hematology Department Hospital de la Santa Creu i Sant Pau Barcelona Spain; ^2^ Hematology Department Institut Català d’Oncologia Hospital Duran i Reynals. Hospitalet de Llobregat Barcelona Spain

**Keywords:** chemotherapy, hematological cancer, lymphoma, mutation

## Abstract

**Background:**

Hairy cell leukemia (HCL) is a rare disorder characterized by splenomegaly, pancytopenia, and proliferation with “hairy” lymphocytes. Treatment is based on purine analogs and anti‐CD20 antibodies, often resulting in significant adverse effects.

**Aims:**

The objective of this study is to describe the frequency, clinical, and biological characteristics of a historic cohort of HCL patients in our center and the most common side effects related to treatment with purine analogs.

**Methods and Results:**

This study analyzed 21 patients treated between 2009 and 2023, focusing on clinical characteristics, treatment response, complications, and survival outcomes. Cladribine treatment achieved complete response in 77.8% of patients. The 5‐year OS and PFS were 100% and 91.7%, respectively. Infections, pathogens such as herpes viruses and mycobacteria, were major complications, impacting 38% of patients. Severe skin reactions were noted in patients treated with cladribine.

**Conclusion:**

The study highlights cladribine's effectiveness in inducing remission in HCL patients, pointing out the significant risks of infections and other adverse effects. Introducing targeted treatments like BRAF inhibitors provides promising alternatives, especially for resistant patients or those intolerant to purine analogs. Future strategies should focus on integrating targeted therapies to reduce treatment‐related morbidity.

## 1 Introduction

Hairy cell leukemia (HCL) is an infrequent chronic lymphoproliferative neoplasia that is characterized by an indolent course, marked splenomegaly, progressive pancytopenia in many cases, and rare circulating tumoral cells, usually with no lymphadenopathy [1]. Currently, the treatment of HCL is based on purine analogs and in combination with anti‐CD20 monoclonal antibodies [2, 3], which induce durable remissions for most patients.

However, infectious complications remain a significant issue in these patients [4, 5].

The same dilemma applies to HCL‐variant (HCL‐v), now reclassified within the splenic B‐cell lymphoma/leukemia with prominent nucleoli category and some cases of B‐cell prolymphocytic leukemia. This reclassification emphasizes that it is not merely a “variant” of classic HCL but a separate disease with its own diagnostic criteria [6, 7]. It represents 10% of all HCL cases, and aside from the low HCL score [8, 9], it can be differentiated by the absence of BRAF V600E mutation [10].

Recent understanding and advances in therapies in other chronic lymphoproliferative neoplasia have also translated into excellent results in HCL [11]. One critical unmet need was the reduction of morbidity of therapies, mainly due to off‐target toxicity on effector and regulatory cells of the immune system.

HCL‐v have dismal prognoses, but recently promising results with ibrutinib monotherapy have been reported in second line [12].

The BRAF V600E mutation is present in a large part of the HCL and downstream MEK–ERK signaling pathway, leading to malignant B‐cell proliferation. Patients who lack BRAF V600E may harbor alternative BRAF mutations [13], and in the future, it will be essential to understand the tumoral microenvironment of HCL with mutated or unmutated BRAF V600E.

## 2 Materials and Methods

In this retrospective unicentric study, we collected demographic and clinical information from 21 patients diagnosed with HCL in our institution from 2009 to 2023 (18 patients with treatment indication and two without treatment).

The collected variables were sex, age at diagnosis, time to treatment initiation, tumoral immunophenotype, BRAF mutation status, treatment response, treatment complications, time to relapse, status at last visit, and cause of death.

A complete remission is defined as near normalization of peripheral blood counts (hemoglobin > 11 g/dL, platelets > 100 × 10^9^/L, and neutrophils > 1.5 × 10^9^/L) along with regression of splenomegaly and morphological absence of hairy cells in the bone marrow and peripheral blood [14].

Progression‐free survival (PFS) was calculated from the date of diagnosis until the date of relapse. Overall survival (OS) was calculated from the date of diagnosis to the date of the last follow‐up. Survival analysis was performed using Kaplan–Meier, and differences were determined using the log‐rank test (*p* < 0.05). Statistical analysis was performed using IBM SPSS 25 statistical software.

We aimed to describe the frequency, clinical, and biological characteristics of HCL patients in our center and the most common side effects related to treatment with purine analogs.

Our institution's ethical committee board approved the study.

## 3 Results

Table 1 summarizes the baseline characteristics of the study population (*N* = 21). There was a male predominance (57%), and the median age at diagnosis was 61. Most cases were HCL, and only three patients were HCL‐variant when using Matute's score. Patients with treatment indication (*n* = 19) were mainly treated with purine analogs (Cladribine 0.12 mcg/kg/day, for 5 consecutive days); only one patient underwent splenectomy.

**TABLE 1:** Baseline characteristics at diagnosis (*n* = 21).

	Mean	Median
Age	61.7	61.0
Hemoglobin (g/dL)	12.3	12.5
Platelets (×10^9^/L)	126.3	100.0
Leukocytes (×10^9^/L)	4.0	2.9
Lymphocytes (×10^9^/L)	1.9	1.3
Neutrophils (×10^9^/L)	1.7	1.1
LDH	238.8	219.5

	Frequency	Percentage
*BRAF status*		
Mutated	7.0	33.30%
Wild type	8.0	38.10%
Not evaluable	5.0	23.80%
Not assessed	1.0	4.80%
*CD*38 (*n* = 14)		
Positive	6	42.90%
*CD*22 (*n* = 21)		
Positive	21	100%
*CD*11*c* (*n* = 21)		
Positive	21	100%
IGHV status		
Mutated	1.0	4.80%
Wild type	5.0	23.80%
Not assessed	15.0	71.80%
*Matute's score* > 3	18	85.70%
*Cytogenetics* (*n* = 12)		
Normal	11.0	91.60%
Complex	1.0	8.60%
*Gender*		
Male	12.0	57%
Female	9.0	43%
*Splenomegaly*	14	67%

Splenomegaly is very common at the time of diagnosis (67%), and the most relevant cytopenia was thrombocytopenia (median 100 × 10^9^/L (17–333 × 10^9^/L)). Flow cytometry shows strong CD22, CD20, and CD11c expression, and CD38 was positive in 43% of patients.

Our study found BRAF mutation in 33% of assessed patients. Around 25% of HCL patients had unmutated IGHV, although it was not tested in more than half of the patients, which is the main limitation of our study, as well as detecting BRAF mutation in only one‐third of the patients.

Eighteen patients received Cladribine (2CDA) treatment in the first line. Eleven patients (61%) required one cycle of 2CDA, and seven patients (41%) required two cycles to achieve a response. Fourteen patients (77.8%) achieved a complete response and four (22.2%) achieved a partial response after treatment. After a median follow‐up of 51 months (6–170 months), three cases of the cohort relapsed, and one case died due to other comorbidities.

The 5‐year OS and PFS were 100% and 91.7% (95% CI, 77.3%–100%), respectively.

**FIGURE 1:**
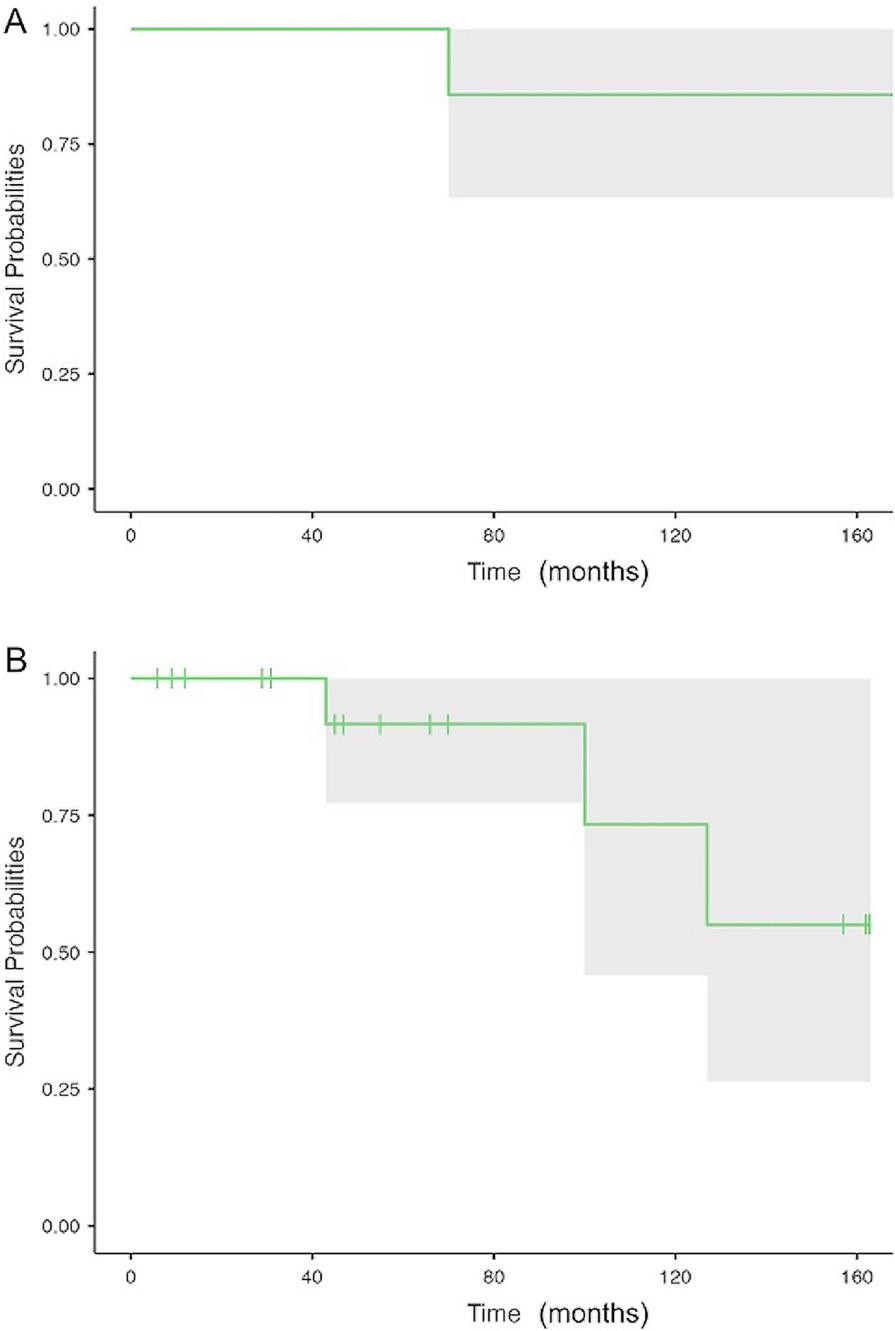
Overall survival (A) and progression‐free survival (B) of 2‐CDA‐treated patients.

Figure 1 shows the cohort's OS and PFS.

Only one patient in the cohort died due to disseminated colorectal cancer.

In our cohort, infections were identified in 38% of patients during the follow‐up period (Table 2). The most frequently isolated microorganisms were from the herpes virus family and mycobacteria. Of the patients, 80% and 67% received prophylaxis against *Pneumocystis Jirovecii* and herpes simplex virus, respectively.

**TABLE 2:** Infections.

Case 1	*Candidiasis* sp.
Case 2	*Escherichia coli* bacteremia
Case 3	*Herpes simplex* virus
Case 4	*Varicella‐zoster* virus, Sars‐Cov‐2 pneumonia, *Influenza A* virus
Case 5	*Mycobacterium bovis* bacteremia
Case 6	*Mycobacterium tuberculosis*
Case 7	*Staphilococcus haemolyticus* bacteraemia

There were four cases of severe skin reactions and multiple drug hypersensitivity due to T‐cell imbalance induced by 2CDA. All reactions were treated with antihistamines and corticosteroids [4].

## 4 Discussion

Hairy cell leukemia (HCL) is a rare hematological malignancy characterized by mature lymphocytes with “hairy” projections in the bone marrow and spleen. It is less common in peripheral blood. At the time of diagnosis, patients frequently present with pancytopenia and splenomegaly, and the increased risk of infections is characteristic throughout the disease course [15].

BRAF V600E mutation is detected in the vast majority of cases of HCL. Patients whose mutation is not identified may have cases of v‐HCL or alternative BRAF mutations. Mutations of TP53 can be detected in a small proportion of patients and are associated with an unmutated IGHV status, resistance to cladribine, and shorter event‐free survival [14]. In our cohort, in 38% of patients, BRAF V600E mutation was not detected, but in up to 28.6% of cases, either detection was not assessed or it was not evaluable due to the low quality of DNA extracted from paraffin‐embedded blocks, which was the main limitation of our study. In these cases, BRAF immunohistochemistry would have been useful, but it was not available in our center at the time of diagnosis. In fact, the BRAF V600E mutation was not integrated into routine diagnostic work‐up guidelines until approximately 2017. Consequently, cases diagnosed before this time were classified based on classic morphological and immunophenotypic criteria alone.

We acknowledge that the small sample of our series is a limitation, but we believe it still provides valuable insights. We plan for a multi‐center follow‐up study.

Purine analogs are indicated in first‐line HCL patients, conferring in most cases a long OS, as it is shown in our study. However, combining chemoimmunotherapy as a first‐line treatment represents an increasingly used therapeutic approach, and recent data suggest that it could improve the duration of the complete response.

Purine analogs prolong the suppression of immune effector cells (e.g., CD41 T cells), increasing the risk of opportunistic infections, which are the leading causes of death [14].

The previously observed predisposition of patients with HCL to infections is confirmed in our cohort, where the infection rate was 38%, especially infections due to mycobacteria, the herpes virus family, or respiratory viruses, and two cases of bacteremia by gram‐negative and coagulase‐negative staphylococcus. The institutional protocol for patients receiving purine analogs was updated in 2015 due to the recognized risk of *Pneumocystis jirovecii* pneumonia (PJP) and included the systematic prophylactic use of TMP/SMX and acyclovir. Patients treated before this date did not routinely receive PJP prophylaxis, while those treated after 2015 did.

Kapoor et al. describe the incidence and characteristics of infections in a retrospective cohort with 149 patients. The infectious rate (36.2%) is comparable to that observed in our study. Most cases (69%) were bacterial; secondly, they observed viral infections, including viruses of the respiratory tract and reactivation of the varicella‐zoster virus, as it shown in our study, and finally, fungal infections [5].

Health education strategies for infection prevention and supportive treatments, such as anti‐infective prophylaxis or myeloid growth factors, are essential in managing these patients. Using new targeted drugs is another way to reduce the risk of infections, and moving forward with these strategies will impact infection control.

BRAF inhibitors (Vemurafenib) are used in patients in whom purine analogs are contradicted, such as patients with serious infections or refractory conditions. Responses have been shown both in monotherapy and in combination with Rituximab, with a high overall response rate (ORR) (96%–100%) and complete response rate (> 87%) [16]. It is essential to recognize side effects from BRAF inhibitors, such as skin rash, arthritis, or secondary skin tumors.

Vemurafenib plus Obinutuzumab has been tested for untreated classical HCL in a multi‐center, open‐label, single‐arm phase II study (NCT03410875). In this trial, it was shown that combined time‐limited Vemurafenib and Obinutuzumab achieved complete response in more than 90% of patients, and no patient experienced disease relapse at a median follow‐up of 34.9 months [17].

The novel therapy Moxetumomab pasudotox (HA22, CAT‐8015) is an immunotoxin directed against a specific cell surface target, such as CD22 [18]. It was available for relapsed/refractory HCL patients with or without BRAFV600E mutations and v‐HCL; nevertheless, it is currently not used since it was withdrawn from the market.

On the other hand, a higher incidence of antibiotic allergies has been described in patients treated with 2CDA compared to patients not treated or treated with other drugs. This complication probably occurs due to immune dysregulation in the setting of profound CD4+ lymphopenia caused by 2CDA [4]. Our cohort detected 4 cases of allergies and skin reactions related to 2CDA.

The development of secondary neoplasms has also been reported in the literature as a treatment‐related complication, and age and cumulative treatment are probably the most important risk factors. Another key consideration in these patients is whether there is an inherent predisposition to secondary neoplasms due to immune dysfunction associated with HCL. In this respect, further investigation is still needed.

Based on the high morbidity of infections in these patients and the reasonable response rates of new targeted drugs, it would be interesting to introduce targeted therapies in previous lines in refractory patients and especially in those with an increased risk of infection.

## 5 Conclusion

The study highlights cladribine's effectiveness in inducing remission in HCL patients, pointing out the significant risk of infections and other adverse effects. Introducing targeted treatments like BRAF inhibitors provides promising alternatives, especially for resistant patients or those intolerant to purine analogs. Future strategies should focus on integrating targeted therapies to reduce treatment‐related morbidity.

## Author Contributions

**M. Garcia Fasanella:** conceptualization (equal), data curation (lead), formal analysis (equal), writing – original draft (lead), writing – review and editing (equal). **A. Mozos:** data curation (supporting), methodology (supporting), project administration (equal), validation (equal). **J. Briones:** project administration (supporting), validation (supporting), writing – review and editing (supporting). **J. F. Nomdedeu:** methodology (supporting), project administration (equal), supervision (equal), validation (equal). **S. Novelli:** conceptualization (equal), formal analysis (equal), methodology (equal), supervision (equal), validation (equal), writing – review and editing (supporting).

## Ethics Statement

The study was approved by the ethics committee (CEIm Sant Pau Campus Salut Barcelona).

## Consent

Informed consent was obtained from all participants of this study.

## Conflicts of Interest

The authors declare no conflicts of interest.

## Data Availability

The data that support the findings of this study are openly available in Pubmed and Figshare at (https://pubmed.ncbi.nlm.nih.gov/) and (https://figshare.com/), respectively.

## References

[1] E. Tiacci , V. Trifonov , G. Schiavoni , et al., “BRAF Mutations in Hairy‐Cell Leukemia,” New England Journal of Medicine 364, no. 24 (2011): 2305–2315. 21663470 10.1056/NEJMoa1014209 PMC3689585

[2] R. Belani and A. Saven , “Cladribine in Hairy Cell Leukemia,” Hematology/Oncology Clinics of North America 20, no. 5 (2006): 1109–1123. 16990111 10.1016/j.hoc.2006.06.008

[3] D. Chihara , E. Arons , M. Stetler‐Stevenson , et al., “Long Term Follow‐Up of a Phase II Study of Cladribine With Concurrent Rituximab With Hairy Cell Leukemia Variant,” Blood Advances 5, no. 23 (2021): 4807–4816. 34607348 10.1182/bloodadvances.2021005039 PMC9153043

[4] Z. Meher‐Homji , C. S. Tam , J. Siderov , et al., “High Prevalence of Antibiotic Allergies in Cladribine‐Treated Patients With Hairy Cell Leukemia–Lessons for Immunopathogenesis and Prescribing,” Leukemia & Lymphoma 60, no. 14 (2019): 3455–3460. 31256738 10.1080/10428194.2019.1633640 PMC6928424

[5] N. Kapoor , Q. Zhao , A. Stiff , et al., “Incidence, Description, and Timing of Serious or Opportunistic Infections in Patients With Hairy Cell Leukemia Treated With Purine Nucleoside Analogues,” Blood 140 (2022): 9361–9362, 10.1182/blood-2022-156720.

[6] E. Campo , E. S. Jaffe , J. R. Cook , et al., “The International Consensus Classification of Mature Lymphoid Neoplasms: A Report From the Clinical Advisory Committee,” Blood 140, no. 11 (2022): 1229–1253, 10.1182/blood.2022015851. 35653592 PMC9479027

[7] R. Alaggio , C. Amador , I. Anagnostopoulos , et al., “Correction: “the 5th Edition of the World Health Organization Classification of Haematolymphoid Tumours: Lymphoid Neoplasms” Leukemia. 2022, 36(7): 1720–1748,” Leukemia 37, no. 9 (2023): 1944–1951, 10.1038/s41375-023-01962-5. 35732829 PMC9214472

[8] E. Matutes , R. Morilla , K. Owusu‐Ankomah , et al., “The Immunophenotype of Hairy Cell Leukemia (HCL). Proposal for a Scoring System to Distinguish HCL From B‐Cell Disorders With Hairy or Villous Lymphocytes,” Leukemia & Lymphoma 14, no. S1 (1994): 57–61, https://www.semanticscholar.org/paper/87feb0a7daac605240ba407556857f2af4500ae5. 7820054

[9] M. Stetler‐Stevenson and P. R. Tembhare , “Diagnosis of Hairy Cell Leukemia by Flow Cytometry,” Leukemia & Lymphoma 52, no. S2 (2011): 11–13. 21504292 10.3109/10428194.2011.570820

[10] T. Robak , E. Matutes , D. Catovsky , P. L. Zinzani , C. Buske , and ESMO Guidelines Committee , “Hairy Cell Leukaemia: ESMO Clinical Practice Guidelines for Diagnosis, Treatment and Follow‐Up,” Annals of Oncology: Official Journal of the European Society for Medical Oncology 26, no. S5 (2015): v100–v107, 10.1093/annonc/mdv200. 26269205

[11] E. Maitre , J. Paillassa , and X. Troussard , “Novel Targeted Treatments in Hairy Cell Leukemia and Other Hairy Cell‐Like Disorders,” Frontiers in Oncology 12, no. December (2022): 1–14. 10.3389/fonc.2022.1068981 PMC9815161 36620555

[12] K. Rogers , E. M. McLaughlin , L. Wei , et al., “Extended Follow Up of a Phase 2 Study of Ibrutinib in Hairy Cell Leukemia,” Blood 140 (2022): 6494–6495, 10.1182/blood-2022-165795.

[13] S. Tschernitz , L. Flossbach , M. Bonengel , S. Roth , A. Rosenwald , and E. Geissinger , “Alternative BRAF Mutations in BRAF V600E‐Negative Hairy Cell Leukaemias,” British Journal of Haematology 165 (2014): 529–533. 24433452 10.1111/bjh.12735

[14] M. R. Grever , O. Abdel‐Wahab , L. A. Andritsos , et al., “Consensus Guidelines for the Diagnosis and Management of Patients With Classic Hairy Cell Leukemia,” Blood 129, no. 5 (2017): 553–560. 27903528 10.1182/blood-2016-01-689422 PMC5290982

[15] A. Mendez‐Hernandez , K. Moturi , V. Hanson , and L. A. Andritsos , “Hairy Cell Leukemia: Where Are we in 2023?,” Current Oncology Reports 25, no. 8 (2023): 833–840. 37097545 10.1007/s11912-023-01419-z PMC10126561

[16] E. Tiacci , J. H. Park , L. De Carolis , et al., “Targeting Mutant BRAF in Relapsed or Refractory Hairy‐Cell Leukemia,” New England Journal of Medicine 373 (2015): 1733–1747. 26352686 10.1056/NEJMoa1506583 PMC4811324

[17] J. H. Park , S. Devlin , B. H. Durham , et al., “Vemurafenib and Obinutuzumab as Frontline Therapy for Hairy Cell Leukemia,” NEJM Evidence 2, no. 10 (2023): EVIDoa2300074. 38320179 10.1056/EVIDoa2300074 PMC11110928

[18] J. Paillassa , E. Maitre , and X. Troussard , “Hairy Cell Leukemia (HCL) and HCL Variant: Updates and Spotlights on Therapeutic Advances,” Current Oncology Reports 24 (2022): 1133–1143. 35403971 10.1007/s11912-022-01285-1

