# Correction to “Evaluation of Clinical Outcomes and Treatment Complications in Hairy Cell Leukemia: A Single‐Center Retrospective Analysis”

**DOI:** 10.1002/cnr2.70392

**Published:** 2025-11-19

M. Garcia Fasanella, A. Mozos, J. Briones, J. F. Nomdedeu, and S. Novelli, “Evaluation of Clinical Outcomes and Treatment Complications in Hairy Cell Leukemia: A Single‐Center Retrospective Analysis,” *Cancer Reports* 8, no. 10 (2025): e70356, https://doi.org/10.1002/cnr2.70356.

The authors would like to add an Acknowledgments section to the above‐mentioned article. The Acknowledgments section is shown below:

**Acknowledgments**

We thank CERCA Programme/Generalitat de Catalunya for institutional support.

We apologize for this error.

